# Safe use of contrast media in myasthenia gravis: systematic review and updated European Society of Urogenital Radiology Contrast Media Safety Committee guidelines

**DOI:** 10.1007/s00330-023-10463-z

**Published:** 2023-12-14

**Authors:** Remy W. F. Geenen, Giles Roditi, Marie-France Bellin, Michele Bertolotto, Torkel Brismar, Jean-Michel Correas, Ilona A. Dekkers, Gertraud Heinz-Peer, Andreas H. Mahnken, Aart J. van der Molen, Carlo C. Quattrocchi, Alexander Radbuch, Peter Reimer, Maria Sebastià, Fulvio Stacul, Laura Romanini, Olivier Clément, on behalf of the Contrast Media Safety Committee

**Affiliations:** 1 Department of Radiology, Northwest Clinics, Alkmaar, The Netherlands; 2 Department of Radiology, Glasgow Royal Infirmary, Glasgow, UK; 3 BioMaps, Service de Radiologie, University Hospital Bicêtre, AP-HP, University Paris-Saclay, Le Kremlin-Bicêtre, France; 4 Department of Radiology, University Hospital Trieste, Trieste, Italy; 5 Unit of Radiology, Department of Clinical Science, Intervention and Technology, Karolinska Institutet, Stockholm, Sweden; 6 Department of Radiology, Karolinska University Hospital in Huddinge, Stockholm, Sweden; 7 Service de Radiologie, DMU Imagina, Groupe Hospitalier Necker, AP-HP, Université de Paris-Cité, Paris, France; 8 Department of Radiology, Leiden University Medical Center, Leiden, The Netherlands; 9 Department of Radiology, Landesklinikum St Pölten, St Pölten, Austria; 10 Department of Diagnostic and Interventional Radiology, Marburg University Hospital, Marburg, Germany; 11 Centre for Medical Sciences-CISMed, University of Trento, Via S. Maria Maddalena 1, 38122 Trento, Italy; 12 Clinic for Diagnostic and Interventional Neuroradiology, University Clinic Bonn, Bonn, Germany; 13 German Center for Neurodegenerative Diseases, DZNE, Bonn, Germany; 14 Department of Radiology, Institute for Diagnostic and Interventional Radiology, Klinikum Karlsruhe, Karlsruhe, Germany; 15 Department of Radiology, Hospital Clinic de Barcelona, Barcelona, Spain; 16 Department of Radiology, Ospedale Maggiore, Trieste, Italy; 17 Department of Radiology, ASST Cremona, Cremona, Italy; 18 Service de Radiologie, DMU Imagina, Hôpital Européen Georges Pompidou, AP-HP, Université Paris-Cité, 20 Rue LeBlanc, 75015 Paris, France

**Keywords:** Myasthenia gravis, Contrast media, Multidetector computed tomography

## Abstract

**Objectives:**

It is uncertain whether modern iodine-based or gadolinium-based contrast media (CM) administration can lead to increased symptoms in patients with myasthenia gravis.

**Methods:**

A systematic search in Medline was conducted for studies describing the symptomatology of myasthenia gravis patients before and after receiving intravenous (IV) CM and having a matched control group of myasthenia gravis patients who did not receive IV CM.

**Results:**

Three retrospective studies were selected with a total of 374 myasthenia gravis patients who received iodine-based CM and a total of 313 myasthenia gravis patients who underwent unenhanced CT and served as controls. Pooling of the data from the three retrospective studies showed that in 23 of 374 patients, increased symptoms after iodine-based CM administration were described (6.1%). Increased symptomatology also occurred in 11 of 313 patients after unenhanced CT (3.5%). When looking more deeply into the data of the three studies, conflicting results were found, as two articles did not find any relationship between CM and myasthenia gravis symptoms. The remaining study only found a significant increase in symptomatology within 1 day after CT scanning: seven patients (6.3%) in the contrast-enhanced CT group and one patient (0.6%) in the unenhanced CT group (*p* = 0.01).

**Conclusions:**

There is limited evidence on the relationship between CM and myasthenia gravis symptoms. In the vast majority of myasthenia gravis patients, CM are safe. Probably, in less than 5% of the patients, iodine-based CM administration may lead to increased severity of the symptoms within the first 24 h after administration.

**Clinical relevance statement:**

Be aware that intravenous administration of iodine-based contrast media can lead to an increase of symptoms in patients with myasthenia gravis within the first 24 h. This can probably happen in less than 5% of the patients.

**Key Points:**

*• It is unclear whether modern contrast media can lead to increased symptoms in myasthenia gravis patients after intravenous administration.*

*• There seems to be a small risk of increased myasthenia gravis symptoms within 24 h after intravenous administration of iodine-based contrast media, probably in less than 5% of the administrations.*

*• Gadolinium-based contrast media are safe for patients with myasthenia gravis.*

## Introduction

Myasthenia gravis is an autoimmune disease in which antibodies bind to acetylcholine receptors or functionally related molecules in the postsynaptic membrane at the neuromuscular junction [1]. These antibodies induce weakness of skeletal muscles, the sole disease manifestation. Patients are defined on the basis of autoimmune and antibody disease mechanisms, target molecules of skeletal muscle, thymic status, genetics, response to therapy, and disease phenotype into seven different subgroups, including the thymoma subgroup, which constitutes 10% of the patients [1]. The annual incidence is eight to ten cases per 1 million persons with a prevalence of 150–250 cases per million, making it the major disease that affects the neuromuscular junction [1].

In the era of ionic high-osmolar contrast media (CM), several case reports and small series without control groups described adverse outcomes and myasthenic crisis in patients with myasthenia gravis who received iodine-based CM [2–8]. There is also one case report from 1992 on acute deterioration of myasthenia gravis after intravenous administration (IV) of a linear gadolinium-based CM [9]. The possible mechanism of CM leading to myasthenic crisis is unknown, although it has been postulated that CM might interfere with the calcium-mediated acetylcholine release [10].

The question remains whether in the era of non-ionic low- and iso-osmolar iodine-based CM and in the era of more stable cyclic Gd chelates acute deterioration of myasthenia gravis symptoms by CM still exists. Therefore, the European Society of Urogenital Radiology (ESUR) Contrast Media Safety Committee (CMSC) considered it necessary to undertake a systematic review of the available literature about CM and myasthenia gravis in order to provide up-to-date guidance. Special emphasis was to find articles with control groups of myasthenia gravis patients who did not receive IV CM and whose symptoms were recorded for the research period. The results of this review and the following guidelines were extensively discussed by the CMSC academic members and were also reviewed by representatives of the four major contrast media companies (Bayer, Bracco, GE, and Guerbet), who are consultants to the CMSC. Both academic members and representatives had been sent a version of this article for review in advance of the June 2022 meeting. During the meeting, everybody could ask questions and provide suggestions to improve the guidance. The academic members agreed on the final version by consensus. This was achieved at their meeting in June 2022.

## Methods

A systematic search in Medline was performed for studies reporting deterioration of myasthenia gravis symptomatology, the preceding administration of CM, and a control group of myasthenia patients who did not receive IV contrast media. A combination of Medical Subject Headings (MeSH) terms contrast media and myasthenia gravis was used. The MeSH term contrast media also encompasses other entry terms such as contrast agent, contrast material, radiocontrast media, radiocontrast agent, and radiopaque media. The MeSH term myasthenia gravis also encompasses other entry terms of clinical and receptor subtypes. A total of 15 articles were found. After reading the title and abstract, articles were selected when they described patients with myasthenia gravis who received IV CM and a control group of myasthenia patients who did not receive CM existed. Based on these criteria, three articles were selected by one reviewer (R.G.), with 24 years of experience in diagnostic radiology. Two articles described retrospective series on myasthenia gravis patients who underwent contrast-enhanced CT (CECT) and a control group of myasthenia gravis patients that underwent CT without contrast [11, 12]. The third article has the same research design, but patients who underwent MRI were also included [13].

Of these three articles, in Medline the similar article option was used. This option creates a list of articles by comparing words from the title, abstract, and Mesh terms using a word-weighted algorithm without the use of filters. This led to 240 additional articles on the article of Somashkehar et al [11], 83 additional articles on the article of Rath et al [12], and 80 additional articles on the article of Mehrizi et al [13]. The titles of all these additional articles were read and if necessary and applicable, the abstract was read by one reviewer (R.G.). This led to three additional papers in the form of recent case reports on this subject [14–16]. As literature on myasthenia gravis and CM is limited, these three case reports on three patients were also included, as they provide some additional views on this subject.

Based on the literature and the CMSC consensus, the guideline was graded using the Oxford Centre for Evidence Based Medicine (OCEBM) 2011 evidence classification: level 1: systematic review of randomized trials, level 2: randomized trial, level 3: non-randomized controlled cohort/follow-up study, level 4: case series, case-controlled, or historically controlled studies, level 5: mechanism-based reasoning [17].

## Results

Somashekar et al describe 112 myasthenia gravis patients undergoing CECT and 155 myasthenia gravis patients undergoing unenhanced CT in the period 1995–2011 [11]. Statistics were performed with the Mantel–Cox log rank test, chi-square test, or Fisher exact test where appropriate. Comparison of both groups did not show any significant differences when it came to demographics, medical history, myasthenia gravis symptomatology immediately before CT, and medical therapy for myasthenia gravis. There was a significant difference in CT indication, with unenhanced CT being performed more often for acute neuromuscular compromise and CECT being performed more often for acute dyspnea (*p* = 0.002). In the CECT group, several non-ionic CM were used and the specific type could not be traced in the records in 54 of 112 patients (48%). Disease-related symptom exacerbation within 45 days after the index CT scan occurred in 14 CECT patients (12.5%) and in nine patients (5.8%) in the unenhanced CT group. Both groups had a similar proportion of new or worsening symptoms occurring 2–7 days (*p* = 0.70) and 8–45 days (*p* = 0.99) after the index CT. Between 2 and 7 days, this occurred in a total of six patients, three patients in each group. Between 8 and 45 days, this occurred in a total of nine patients, four in the CECT group and five in the unenhanced CT group. However, within the first day after the index CT, the CECT group had a significantly higher rate of acute symptoms (*p* = 0.01) (6.3% [seven of 112 patients; 95% confidence interval 0.03, 0.12] vs. 0.6% [one of 155 patients; 95% confidence interval 0.0002, 0.04]). Six of seven patients developed new respiratory symptoms; the 7th patient developed progressive weakness. Of the 112 patients in the CECT group, 66 (59%) had stable symptoms, 17 (15%) had deteriorating symptoms, and 29 (26%) had only recently diagnosed with myasthenia gravis. Two patients in the stable group, two in the deteriorating group, and three in the just diagnosed group showed symptom exacerbation within 1 day after CECT. Five of the seven patients received medical therapy at time of the CECT; the other two started with medical therapy after the CT. In the unenhanced CT group of 155 patients, symptoms of 83 (54%) patients were stable, symptoms were deteriorating in 34 (22%) patients, and 38 (25%) patients had been recently diagnosed with myasthenia gravis. The only case of symptom exacerbation happened in the just diagnosed group and this patient had already started medical therapy before the unenhanced CT. The time of onset to exacerbation was significantly shorter in the CECT group (2.5 days) than in the unenhanced CT group (14 days) (*p* = 0.05). The authors conclude that a significant association between intravenous low-osmolality CM and acute exacerbation within 1 day after administration of myasthenia gravis symptoms exists, with an incremental frequency that is 5–6% above baseline [11].

Rath et al report on 73 myasthenia gravis patients who underwent CECT with low-osmolality CM and 52 patients who underwent unenhanced CT in the period 2005–2015 [12]. Statistics were performed with the Man-Whitney *U* test, Student’s *t* test, the chi-square test, or the Fisher exact test where appropriate. Furthermore, univariate and multivariate logistic regression analyses were used to compare the primary endpoint, clinically relevant deterioration of myasthenic symptoms within 30 days of the CT study, between the two groups. The baseline characteristics were well matched between both groups as well as the disease duration, antibody status, and current medical therapy. The two groups differed in the CT indication and body region scanned. Scans of the chest and abdomen were performed more often with CM in comparison with scans of the head and other regions whereas dyspnea and acute, non-neurological symptoms were indications for CECT. At least two different non-ionic low-osmolar CM were used and in 45 of 73 patients (62%), the exact type of CM could not be extracted retrospectively. Nine patients in the CECT group (12.3%) and two patients in the unenhanced CT group (3.8%) had worsening of their symptoms within 30 days of the CT (*p* = 0.12) (odds ratio 3.52, 95% CI 0.73 17.0) The mean time to worsening was 11.1 days in the CECT group and 13 days in the unenhanced CT group. The medical records of the nine CECT patients were critically reviewed and authors concluded that in none of these patients were CM a likely cause of the deterioration [12].

Mehrizi et al reported on 354 myasthenia gravis patients, of whom 189 underwent CECT, 106 unenhanced CT, 42 CEMRI, and 17 unenhanced MR in the time period 2001–2012 [13]. Two different types of non-ionic low-osmolar CM were used. For MRI, one cyclic Gd chelate or a liver-specific linear Gd chelate was used. One patient from the CECT group reported nausea and vomiting. No worsening of the myasthenia symptoms occurred in any of the groups [13]. This is the only article that addresses gadolinium-based CM and provides the only data that gadolinium-based CM are safe for myasthenia gravis patients. Compared to the other two articles, this article gives a brief description of the patient groups, as no baseline characteristics are described. The demographics of all three studies are summarized in Table 1.

**Table 1:** Summary of the series in literature for iodine-based CM

Author	Contrast-enhanced CT: number of patients	Unenhanced CT: number of patients	Contrast-enhanced CT: mean age	Unenhanced CT: mean age	Exacerbation contrast-enhanced CT group, *N* (%)	Exacerbation unenhanced CT group, *N* (%)
Somashekar et al [11]	112 Men, 57 Women, 55	155 Men, 76 Women, 79	55 years	58 years	14 (12.5%)	9 (5.8%)
Rath et al [12]	73 Men, 31 Women, 42	52 Men, 25 Women, 27	62 years	64 years	9^*^ (12.3%)	2 (3.8%)
Mehrizi et al [13]	189	106	Unknown	Unknown	0	0
Total	374	313			23 (6.1%)	11 (3.5%)

^*^After review by an expert panel, none of the exacerbations was likely due to iodine-based CM

Bonani et al describe a 79-year-old male with suspected myasthenia gravis who underwent a CECT with 200 mL Iohexol 300 mgI/mL [14]. Two hours later, the patient developed acute respiratory failure requiring intubation and ventilation. After 2 days of ventilation, extubation could be performed and the patient was found to have severe muscle weakness in the ear, nose, and throat (ENT) region. While waiting for the acetylcholine-receptor antibody test result, treatment with pyridostigmine was given. This gave a marked improvement in the following 24 h. Later, the acetylcholine-receptor antibody test was positive, confirming myasthenia gravis [14].

A more or less similar case is reported from India [15]. A 41-year-old female with myasthenia gravis and treated with pyridostigmine and prednisolone underwent CECT with 50 mL Iopromide 370 mgI/mL. Immediately thereafter, she developed severe respiratory distress which required intubation and ventilation. She was medically treated with steroids and immunoglobulins and was successfully extubated 3 days later [15].

Recently, a rare case of respiratory failure after a swallow under fluoroscopic guidance of 10 mL of Iohexol resulting in aspiration of contrast into the right main bronchus has been described [16]. This happened to a 48-year-old male patient with myasthenia gravis treated with pyridostigmine, mycophenolate mofetil, and prednisolone. Forty-five minutes after swallowing, the patient had to be intubated and ventilated. A neurologist was consulted and made the diagnosis of myasthenic crisis, probably due to aspiration pneumonitis caused by contrast aspiration. The patient was treated with immunoglobulin and increased doses of his three medications. After 72 h, his respiratory mechanics made remarkable improvement. Due to complications of intubation and ventilation, the patient stayed in the hospital for 18 days [16].

## Discussion

The evidence in the literature on CM and myasthenia gravis is limited, as only three retrospective series with control groups have been published. In a recent consensus guideline, iodine-based CM are mentioned as a class of drugs that should be used with caution in myasthenia gravis patients [18]. Three retrospective series have been published describing a total of 374 patients receiving IV contrast and 313 undergoing either unenhanced CT or MRI, serving as a control group. Pooling of the data from these three retrospective studies showed that in 23 of 374 patients, increased symptoms after iodine-based CM administration were described (6.1%). Increased symptomatology also occurred in 11 of 313 patients after unenhanced CT or MRI (3.5%) [11–13].

When looking more deeply into the data, one article is a short communication on myasthenia gravis symptoms before and after CM administration and does not provide thorough patient characteristics and statistics of the several groups [13]. This only shows that increased symptoms of myasthenia gravis did not occur after IV administration of both iodine-based and gadolinium-based CM in their retrospective groups.

The other two articles describe patient characteristics and statistics in more detail; thus, their results are stronger evidence [11, 12]. However, their comparability is limited, as they looked at partially different patient characteristics. Somashekar et al recorded myasthenia gravis symptomatology immediately before CT scanning and found no significant difference between the CECT and unenhanced CT group [11]. This is an important parameter in interpreting the data and this is lacking in the article of Rath et al [12]. However, Rath et al recorded antibody status and found no significant difference between their groups [12]. Another important parameter, medication, was recorded by both studies [11, 12]. No significant difference existed in medication in the CECT and unenhanced CT groups in both studies. Somashekar et al looked into a period of 45 days after initial CT scan, Rath et al at a period of 30 days. Moreover, a panel of experts went through the charts of the nine patients with increased symptoms after iodine-based IV CM administration in the study of Rath et al and decided that it was highly unlikely that iodine-based CM were responsible for the deterioration. This expert panel is lacking in the study of Somashekar et al who only recorded symptomatology and did not interpret this in the context of the entire clinical picture [11, 12]. Therefore, the percentage of exacerbations due to iodine-based CM administration may be overestimated in this study.

Both studies had, due to their retrospective nature, difficulties in recording the exact type of CM administrated. The exact type, dose, and administration rate were only available in 48% of the patients in the study of Somashekar et al and in 62% of the study of Rath et al [11, 12]. Somashekar et al state that all patients received one of a variety of low-osmolar CM [11]. The study of Rath et al covered the years 2005–2015; thus, all these patients must have received non-ionic CM. Despite the uncertainty of the precise type of CM used in a large group of included patients, we can be sure that all patients received non-ionic CM, and thus, both studies were included as they match the research criteria.

What remains is the significant difference in exacerbations up to 1 day after CT in the study of Somashekar et al [11]. This occurred in seven patients (6.3%) in the CECT group and in one patient (0.6%) in the unenhanced CT group (*p* = 0.01). As no expert panel reviewed these charts, we do not know whether this entire 5–6% difference between both groups is attributable to iodine-based CM. The CMSC panel believes that it is likely that this percentage is an overestimate and that with the limited evidence we have, in daily practice, this figure will be below 5%. Furthermore, the limited data show a trend that symptom exacerbation can happen in patients with both stable and deteriorating or recently diagnosed symptomatology and whether patients are being treated with medication for myasthenia gravis does not necessarily prevent an exacerbation.

Two of the three published case reports show that intravenous non-ionic iodine-based CM can cause an exacerbation of myasthenia gravis [14, 15]. In both publications, the increase in symptomatology occurred within minutes to 2 h after IV CM administration, supporting the detected significant increase in exacerbations within 24 h after IV CM administration noted in the paper of Somashekar et al [11, 14, 15]. The last case report describes a very rare case of an acute exacerbation of myasthenia gravis after aspiration of a non-ionic iodinated CM during a swallow study and it is likely that aspiration pneumonitis of any liquid could have caused this [16].

The data on gadolinium-based CM are even more sparse than on iodine-based CM. Mehrizi et al describe in 42 patients with CEMRI versus 17 patients with unenhanced MRI no increased myasthenia gravis symptomatology [13]. Furthermore, only one case report of increased symptoms after administration of a linear gadolinium-based CM has ever been published [9]. No case reports after cyclic gadolinium-based CM exist.

## Conclusion

Iodine-based CM can cause an aggravation of myasthenia gravis symptoms, although this is rare. The limited literature on this subject shows that these exacerbations usually occur within the first 24 h after IV CM administration. The exact incidence is unknown, probably below 5%.

Gadolinium-based CM, especially cyclic chelates, are safe for myasthenia gravis patients, according to the only study that has looked at that subject and the lack of more than a single case report on this subject. Further research on this subject, in the form of a prospective study of myasthenia gravis patients undergoing CECT with a matched control group of myasthenia gravis patients who did not receive iodine-based CM, is needed in order to clarify when iodine-based CM in myasthenia gravis patients can result in a higher chance of an exacerbation. Guidelines are proposed (Table 2).

**Table 2:** Contrast media and myasthenia gravis: ESUR CMSC guidelines

Guidelines	Level of evidence*
Intravenous administration of low-osmolar and iso-osmolar iodine-based CM can be associated with an exacerbation of myasthenia gravis symptoms, within the first 24 h after administration	4
Exacerbation of myasthenia gravis symptomatology occurs probably in less than 5% of the patients receiving low-osmolar or iso-osmolar iodine-based CM IV	4
Gd-based CM are safe for myasthenia gravis patients	4

Based on three retrospective observational studies [11–13]

^*^Oxford Centre for Evidence Based Medicine (OCEBM) 2011 classification [17]:

Level 1: Systematic review of randomized trials

Level 2: Randomized trial

Level 3: Non-randomized controlled cohort/follow-up study

Level 4: Case series, case-controlled, or historically controlled studies

Level 5: Mechanism-based reasoning

## Abbreviations

CECT: Contrast-enhanced CT
CEMRI: Contrast-enhanced MRI
CM: Contrast media
CMSC: Contrast Media Safety Committee
IV: Intravenous
MeSH: Medical Subject Headings

## Declarations

### Guarantor

The scientific guarantor of this publication is Dr. Remy Geenen.

### Statistics and biometry

Basic statistics.

### Methodology

• retrospective

• observational

• multicenter study
